# Zeolites Reduce the Transfer of Potentially Toxic Elements from Soil to Leafy Vegetables

**DOI:** 10.3390/ma15165657

**Published:** 2022-08-17

**Authors:** Oana Cadar, Zamfira Stupar, Marin Senila, Levente Levei, Ana Moldovan, Anca Becze, Alexandru Ozunu, Erika Andrea Levei

**Affiliations:** 1 INCDO-INOE 2000, Research Institute for Analytical Instrumentation, 67 Donath Street, 400293 Cluj-Napoca, Romania; 2 Faculty of Environmental Sciences and Engineering, Babes-Bolyai University, 30 Fantanele Street, 400294 Cluj-Napoca, Romania

**Keywords:** natural zeolite, soil amendment, potentially toxic element, uptake, leafy vegetable

## Abstract

The ability of natural zeolite amendment to reduce the uptake of potentially toxic elements (PTEs) by lettuce, spinach and parsley was evaluated using pot experiments. PTE concentrations in roots and shoots, as well as the pseudo total (PT), water soluble (WS) and bioavailable (BA) PTE fractions in the amended soils, were assessed. Although the PT PTE concentration was high, the WS fraction was very low (<0.4%), while the BA fraction varied widely (<5% for Cr, Mn and Co, <15% for Ni, Pb and Zn, >20% for Cd and Cu). PTE concentration decreased in both roots and shoots of all leafy vegetables grown on zeolite amended soils, especially at high amendment dose (10%). The uptake of PTEs mainly depended on plant species, PTE type and amendment dose. With the exception of Zn in spinach, the bioaccumulation factor for roots was higher than for shoots. Generally, lettuce displayed the highest PTE bioaccumulation capacity, followed by spinach and parsley. Except for Zn in spinach, the transfer factors were below 1 for all PTEs, all plant species and all amendment doses. Our results showed that the natural zeolites are promising candidates in the reclamation of contaminated soils due to their ability to immobilize PTEs.

## 1. Introduction

Potentially toxic elements (PTEs) spread broadly over the globe, causing severe environmental problems, limiting plant productivity and threatening human health [1,2,3]. Some PTEs, including Cu, Cr and Zn, are considered essential micronutrients at low concentrations, but at high concentrations, they can induce non-carcinogenic hazardous effects in plants, as well as in humans [3]. Most PTEs occur naturally, but due to industrial activities, agricultural practices, urbanization or land use changes are enriched in the different environment compartments and persist for long times due to their nondegradable nature [4,5]. Through contaminated soil and water, the PTEs can bioaccumulate in crops or animal products and, by entering in the food chain, may cause various adverse health effects and pose a serious risk to human health [3,4,6,7].

Vegetables are an essential part of the huma n diet. Leafy vegetables, also known as leafy greens, are short-lived herbaceous plants and represent a popular component of the human diet, being consumed usually raw or with minimum cooking [1]. In case of their cultivation in soils with high PTE concentrations, by irrigation with contaminated waters and industrial effluents, sewage sludge applications or soil fertilization, high levels of PTEs may accumulate in the edible parts of plants, their consumption thus causing potential harmful effects [1,7,8,9,10,11]. 

Various remediation approaches based on mechanical or physio-chemical techniques, such as soil excavation, landfilling, washing and solidification, have been developed to reclaim the soil contaminated by PTEs [9,12]. However, due to their high cost, inefficiency at low PTE concentrations, introduction of secondary pollutants, irreversible changes in soil properties accompanied by the degradation of soil quality, a decrease in productivity and negative consequences on the environment, there is an urgent need to develop efficient, cost-effective and environmentally friendly remediation technologies to reclaim the soil contaminated by PTEs [5,9,12,13]. An effective tool for reducing risks related to the presence of PTEs in soil without significantly altering the natural functions of soil, especially in soil supporting food production, is amendment with different materials (compost, biowaste, lime, biochar) or reactive minerals (zeolites, carbonates, phosphate rocks and clay minerals) [14,15].

Natural zeolites are microporous, crystalline, hydrated aluminosilicates of alkaline or alkaline earth cations with a three-dimensional framework constructed of [SiO_4_]^4−^ and [AlO_4_]^5−^ tetrahedra connected through oxygen bridges [16]. Natural and modified zeolites have received much attention due to their accessibility, non-toxicity, low cost and attractive physicochemical properties (reversible dehydration, adsorption of molecules, ion exchange ability without any structural modification, catalytic activity, etc.). They are extensively used in many areas, such as industry, medicine, cosmetics, agriculture, animal husbandry and pollution control [1,15,17]. The use of natural zeolites as immobilizing agents of PTEs has been also tested [5,14,15].

The remediation of soil through fixation/immobilization techniques does not remove the PTEs but reduces their bioavailability and depends on a large number of factors. Thus, immobilization efficiency has to be evaluated in terms of solubility/leachability and bioavailability. Although the natural zeolites have been widely studied and generally showed a consistent capacity to immobilize PTEs, relevant experiments with natural zeolites and recommended application rates are missing or showed inconsistent results. Some possible explanations could be the special structure and dependence of the cation exchange capacity (CEC) on different factors, including the cage structures, adsorbed ions, structural defects and related gangue minerals [13,18,19]. Consequently, it is important to fill this gap by conducting detailed experiments in order to gather more information intended for generalizing the use of natural zeolites according to a specific soil type and specific crop. The identification of appropriate application rates of immobilizing agents (natural zeolites) to offer a cost-effective and efficient remediation method, to fulfill the green and sustainable remediation principles and to allow PTE immobilization in specific multi-contaminated soil, should be also considered [6,19].

The objective of this research was to investigate the influence of Romanian natural zeolite application on PTE (Cd, Cr, Co, Cu, Mn, Ni, Pb and Zn) mobility in a contaminated agricultural soil from a former mining area and to assess the reduction of PTE uptake by leafy vegetables (lettuce, spinach and parsley) by using different amendment doses.

## 2. Materials and Methods

### 2.1. Chemicals and Plant Materials

All chemicals used were of analytical grade and were purchased from Merck, Darmstadt, Germany. Ultrapure water from a Purelab flex 3 system (Buckinghamshire, UK) was used to dilute the samples and to prepare the standard solutions. The seeds of lettuce (*Lactuca sativa* L., var. *Capitata* “May Queen”), spinach (*Spinacia oleracea* L. var. Matador) and parsley (*Petroselinum crispum* L. var. *Crispum*) produced by Agrosem (Targu Mures, Romania) were purchased from a local specialized store.

### 2.2. Zeolite Collection, Preparation and Characterization

The clinoptilolite-type zeolite was collected from Chilioara quarry located in Northern Romania, ground and sieved to obtain a particle size < 1 mm and thermally treated at 105 °C. The chemical composition, and structural and mineralogical characterization of clinoptilolite-type zeolite has been previously reported [20]. Additionally, the Cd, Cr, Co, Cu, Mn, Ni, Pb and Zn concentrations were determined using an Optima 5300 DV (Perkin-Elmer, Woodbridge, ON, Canada) inductively coupled plasma-optical emission spectrometer (ICP-OES) after microwave digestion using the method described previously [13]. The pH was measured in a 1/5 solid to water (*w/v*) suspension using a Seven Excellence multiparameter (Mettler Toledo, Schwerzenbach, Switzerland) [20]. The cation exchange capacity (CEC) of zeolite was determined by the modified ammonium acetate saturation (AMAS) method reported by Kitsopoulos [21]. The specific surface area (S_BET_) was obtained by nitrogen adsorption and desorption at −196 °C using a Sorptomatic 1990 instrument (Thermo Quest, Milan, Italy). The S_BET_ was estimated using the Brunauer–Emmett–Teller (BET) equation from the linear section of isotherms (0.1 < *p*/*p*_o_ < 0.3), while the pore size distribution was calculated from the desorption branch of the isotherms using the Dollimore–Heal model.

### 2.3. Soil Collection and Characterization

The bulk soil sample was collected from a former mining area in Iara village, Cluj County, Romania (46°33′13″ N and 23°31′04″ E). In the Iara area, the exploitation of precious metals has existed since the Roman times, while in the second half of the 20th century, Fe-bearing skarns, quartz sands and limestone were exploited. Presently, all exploitation activities are ceased, but the mining-related legacy of pollution continues to negatively impact the surrounding environment [13,22]. The surface soil (0–25 cm) used for the experiments was sampled in May 2021 with a stainless-steel shovel and stored in clean paper bags during transport to the laboratory after removal of stones and vegetal residues. For the chemical characterization, an aliquot of the soil sample was air-dried, ground and sieved through a 2-mm sieve before being stored at room temperature. The mean content of soil particles with grain sizes of fine sand (0.2–0.02 mm) and fine dust (0.05–0.002 mm) was the largest, accounting for 40.3% and 26.7% of the total, respectively. The content of dust with grain sizes of 0.02–0.05 mm accounted for 10.7%, while the average content of coarse sand with grain sizes of 2.0–0.02 mm accounted for only 9.6%. In addition, the proportion of clay particles (<0.002 mm) in the soil was 12.7%. Accordingly, the soil used for the experiments belongs to the dusty sandy loam textural class. The loam soil has the advantages of an ideal soil texture type, which is suitable for planting crops, especially vegetables.

The pseudo total (PT) concentration of PTEs (Cd, Cr, Co, Cu, Mn, Ni, Pb and Zn) was extracted in aqua regia (HCl 37%/HNO_3_ 65%, 3/1, *v*/*v*) [13], the water soluble (WS) fraction of the PTEs was extracted in water using a 1/10 soil to water ratio and shaking for 1 h [23], while the bioavailable (BA) fraction of PTEs was extracted in 0.05 M ethylenediaminetetraacetic acid (EDTA) using a 1/5 soil to EDTA ratio and shaking for 1 h [24]. PTE content in the aqua regia extracts was determined by flame atomic absorption spectrometry, while those in the water and EDTA extracts were determined by graphite furnace atomic absorption spectrometry using a PinAAcle 900T (Perkin-Elmer, Shelton, CT, USA) spectrometer and expressed as mg/kg dry weight (*dw*). The pH was measured according to Section 2.2. The cation exchange capacity (CEC) of soil was determined by the NH_4_Cl–NH_4_COOH method described by Ciesielski [25].

### 2.4. Design of the Experiment

Substrates for plant cultivation consisting of PTE-contaminated soil–zeolite mixtures in proportions of 0 (control), 50 g zeolite/kg soil (5%) and 100 g zeolite/kg soil (10%) were prepared in plastic pots (18 cm tall, 16 cm top and 14 cm bottom diameter), watered each second day and left for equilibration for 60 days. The soil moisture content was controlled every two days and maintained approximately at a constant value by weighing the pots. At the end of the equilibration period, soil–zeolite mixtures from each pot sample were collected for the physico-chemical analyses described in Section 2.2 and Section 2.3.

To simulate the adsorption of PTEs by crops under natural environmental conditions, a pot culture experiment was conducted. The contaminated soil–zeolite mixtures were used as substrate to grow three species of leafy vegetables: lettuce (*Lactuca sativa* L., var. *Capitata* “May Queen”), spinach (*Spinacia oleracea* L. var. Matador) and parsley (*Petroselinum crispum* L. var. *Crispum*). We chose these three leafy vegetable species due to their short life cycle, relatively high accumulation of toxic metals and their frequent cultivation around households. The experiment was completely randomized with three experimental treatments, three replications of each and 3 vegetables species. Fifty seeds were placed in Petri dishes containing double-layer filter paper in the upper and lower parts and then placed in a plant growth chamber (Panasonic MLR-352H-PE, Etten-Leur, Netherlands) to accelerate germination (incubation temperature of 25 °C, 12 h/12 h light/dark cycles). Afterwards, ten germinated seeds were placed in each pot. The plants were grown under natural photoperiod at an ambient temperature of 25 ± 2 °C for eight weeks, while being watered with distilled water three-times a week, until the edible parts were completely developed. No additional treatment was applied. At the end of the experiments, the plant material was harvested from each pot and PTE content of roots and shoots was measured.

### 2.5. Plant Collection and Analysis

The leafy vegetables were separated into roots and shoots, washed three times with distilled water, then freeze-dried (FreeZone Benchtop, Labconco, Kansas City, MO, USA) and ground to fine powders. The moisture content of samples was determined by drying the samples to a constant weight at a temperature of 105 °C in a universal oven (UFE 400, Memmert, Germany). The Cd, Cr, Co, Cu, Ni and Pb concentrations in the vegetable samples were determined using graphite furnace atomic absorption spectrometry (GFAAS), while Mn and Zn were determined by flame atomic absorption spectrometry (FAAS) using a PinAAcle 900T (Perkin-Elmer, Shelton, CT, USA) spectrometer after microwave digestion using 5 mL HNO_3_ 65% and 2 mL H_2_O_2_ 30% in a closed-vessel Speedwave Xpert microwave system (Berghof, Eningen, Germany) using the method described previously [8]. Three replicate measurements were carried out for each sample. The calibration standards were prepared from 1000 mg/L single element (Cd, Cr, Co, Cu, Mn, Ni, Pb, Zn) standard solutions (Merck, Germany) by appropriate dilutions. In GFAAS, the calibration standards were prepared by auto-dilution of the highest concentrated standard solutions. Chemical modifiers were used according to the recommendation of the instrument manufacturer. The measured metal concentrations expressed in mg/kg dry weight (*dw*) were converted to mg/kg wet weight (*ww*) considering the moisture of samples, as the maximum levels of Cd and Pb in foodstuffs as regulated by Commission Regulations are expressed in mg/kg *ww* [26,27,28]. The limits of detection were calculated using the calibration parameters (*3sy/x/m*) and considering the sample digestion step, where *sy/x* is the residual standard deviation of the calibration curve, *y* is the intercept and *m* is the slope of the calibration curve [29]. Results obtained were: 0.06 mg/kg for Cd, 0.017 mg/kg for Cr, 0.024 mg/kg for Cu, 0.013 mg/kg for Mn, 0.027 mg/kg for Ni, 0.023 mg/kg for Pb and 0.013 mg/kg for Zn.

### 2.6. Quality Assurance

The accuracy of PTE determination was verified by analyzing the following certified reference materials (CRMs): Potash Feldspar (BCS-CRM 376/1, Bureau of Analyzed Samples, Middlesbrough, UK), Loam Soil (ERM-CC141, Institute for Reference Materials and Measurements, Geel, Belgium) and IAEA-359 Cabbage (International Atomic Energy Agency, Vienna, Austria). Acceptable accuracy (80–120%) and precision (<15%) were obtained in all cases.

### 2.7. Calculation of Bioaccumulation and Transfer Factors

The ability of the natural zeolite to immobilize PTEs in soils and to reduce their bioaccumulation in plants was assessed by calculating the bioaccumulation factor for shoots (BAFs) and for roots (BAFr) according to Equations (1) and (2), as well as the transfer factor (TF) according to Equation (3) [7,30,31,32].

(1) $${\mathrm{BAF}}_{S}=\frac{C_{\mathrm{shoot}}}{C_{\mathrm{soil}}}$$

(2) $${\mathrm{BAF}}_{R}=\frac{C_{\;\mathrm{root}}}{C_{\;\mathrm{soil}}}$$

(3) $$\mathrm{TF}=\frac{C_{\mathrm{shoot}}}{C_{\;\mathrm{root}}}$$

where BAF_S_ is the bioaccumulation factor for shoots; BAF_R_ is the bioaccumulation factor for roots; TF is the transfer factor; C_shoot_ is the PTE concentration in shoots; C_root_ is the PTE concentration in roots; and C_soil_ is the concentration of PTEs in soil found in pseudo total (PT), bioavailable (BA) or water soluble (WS) fractions.

### 2.8. Data Analysis

The differences between PTE concentration in plants grown in soils with different amendment doses was tested by comparing the means of the three replicates using the Tukey test for a significance level of 0.05 using OriginPro (version 2020b) software (OriginLab Corporation, Northampton, MA, USA).

## 3. Results and Discussion

### 3.1. Natural Zeolite

The theoretical CEC value (1980 meq/kg) of the natural zeolite, calculated based on the total content of Na, K, Ca and Mg in zeolite, exceeds the effective CEC value (1450 meq/kg) determined by the AMAS extraction method considering the major extractable cations. The obtained results suggest that almost 75% of exchangeable sites are active, accessible to PTEs found in cationic forms [33]. The specific surface area (S_BET_) of the zeolite is 42 m^2^/g, with a corresponding pore volume of 0.058 cm^3^/g and pore radius of 21 Å. The increase of soil CEC with increasing the amount of zeolite from 625 meq/kg (control, 0) to 789 meq/kg (50 g zeolite/kg soil, 5%) and 797 meq/kg (100 g zeolite/kg soil, 10%) suggests that the addition of zeolite supports the cation exchange. However, the increase of zeolite amendment dose does not determine a significant CEC increase. Consequently, the high CEC value and surface area, and low PTE concentration, make the studied natural zeolite suitable to be used as an amendment for contaminated soils in order to reduce the bioavailability of PTEs.

### 3.2. PTE Concentrations of Soil–Zeolite Mixtures

**Figure 1:**
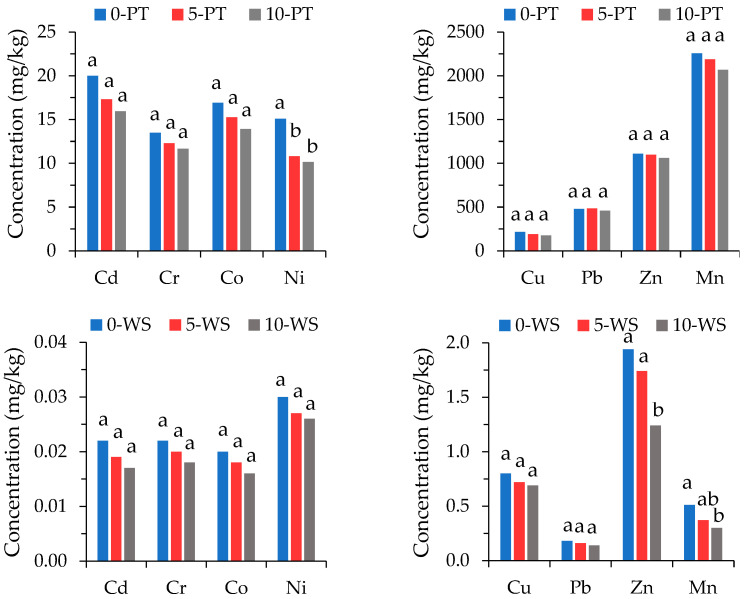

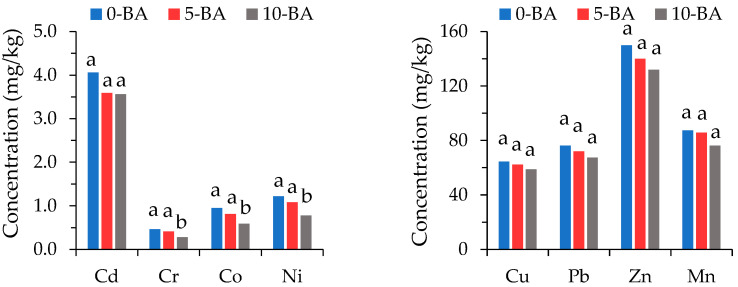
Pseudo total (PT), water soluble (WS) and bioavailable (BA) concentrations of PTEs in the soil–zeolite mixtures in control (0), 50 g zeolite/kg soil amendment (5%) and 100 g zeolite/kg soil amendment (10%). Concentrations of each PTE with different letters are significantly different at *p* < 0.05 significance level.

The concentration of pseudo total (PT), water soluble (WS) and bioavailable (BA) fractions of PTEs in the soil–zeolite mixtures is presented in Figure 1. The PT concentrations of PTEs exceeds the alert thresholds for Mn (1500 mg/kg) and Cu (200 mg/kg) and the intervention thresholds for Cd (5 mg/kg), Cu (200 mg/kg), Pb (100 mg/kg) and Zn (600 mg/kg) according to the Romanian legislation for soil of sensitive use [34]. The PT concentrations of Cr, Co and Ni is below the corresponding alert thresholds for sensitive use. There are no set thresholds for the WS and BA concentrations of PTEs is soils.

As expected, no significant differences is observed between the PT concentrations of PTEs in the amended and unamended soils. Although the PT concentration of PTEs is high, their WS fraction is very low (<2 mg/kg), representing about 0.015–0.40% of the PT concentration. Except for Zn and Mn, no significant differences is found between the WS PTE concentrations in the amended and unamended soils. The BA fraction is low (<1 mg/kg) for Cr and Co, moderate for Ni and Cd (<5 mg/kg) and high for Cu, Mn, Pb and Zn (<150 mg/kg), representing less than 5% of the PT Cr, Co and Mn concentration, less than 15% for Ni, Pb and Zn, around 20% for Cd and 30% for Cu. The difference between the BA fractions is significant for Cr, Co and Ni, but not for the other PTEs. These facts indicate that amendment with zeolite does not change the PT nor the WS fractions, but reduces the bioavailable fraction of some PTEs. A pot experiment conducted to investigate the efficiency of a clinoptilolite rich tuff amendment (0–25% *w*/*w*) for the immobilization of Cd, Zn and Pb in polluted soil resulted in a major decrease in PTE mobility for 0–10% zeolite addition, while at higher doses, the immobilization rate was not considerably improved [33].

**Figure 2:**
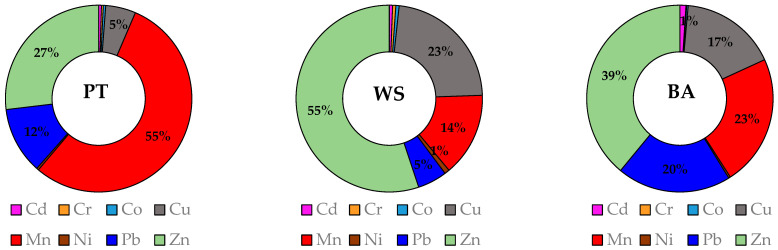
Share of PTEs in the pseudo total (PT), water soluble (WS) and bioavailable (BA) fractions of the control soil (0).

The concentrations of Mn, Zn, Pb and Cu represent more than 97% of the PT PTE concentration in both the amended and unamended soils. In the control soil, the order of PT concentration of PTEs (Mn > Zn > Pb > Cu) changes to Zn > Cu > Mn > Pb in the WS fraction and to Zn > Mn > Pb > Cu in the BA fraction (Figure 2), indicating the presence of Mn species with lower water solubility than that of Zn and Cu and lower bioavailability than that of Zn, Cu and Pb. This fact suggests that Zn species have higher water solubility, as well as higher bioavailability than the Mn species. Thus, in some cases, although the PT PTE contents are high, their availability to plants is low. Oppositely, in some cases, low content of PTEs may have a relatively high percentage of bioavailable fraction that may favor their uptake by plants. Consequently, the bioavailability of PTEs mainly depends on soil conditions, environmental factors, plant species and PTE type [30,35].

### 3.3. PTE Concentration in Plants

The concentrations of PTEs in different vegetables grown on the substrate, consisting of the soil–zeolite mixtures in control (0), 50 g zeolite/kg soil amendment (5%) and 100 g zeolite/kg soil amendment (10%) are given in Table 1.

Generally, a significant decrease in PTE concentration is observed in both roots and shoots of all plant species grown on the amended substrates. However, this difference is much higher in the amendment with 10% zeolite than with 5% of zeolite. The 5% amendment significantly decreases the concentration of Co, Cu, Mn, Pb and Zn in the roots of all studied plant species. The 5% zeolite amendment has no major effect on Cd and Cr content in spinach, but leads to the decrease of the other PTEs in either roots or shoots of spinach. The PTE decrease in the shoots following 5% amendment is observed only for Mn and Zn in spinach, Cd, Co, Cu, Ni and Zn in lettuce and Cr, Cu, Mn, Pb and Zn in parsley. The 10% amendment leads to a significant decrease in all PTEs in both roots and shoots for all elements, except for Cd in the spinach roots.

**Figure 3:**
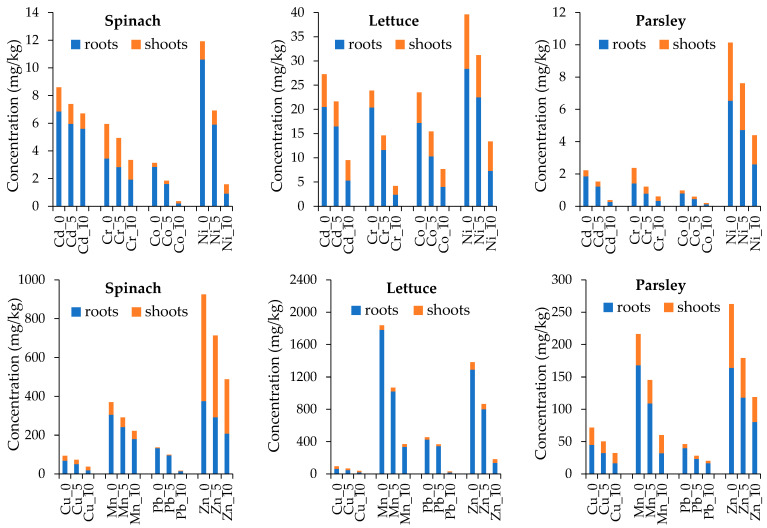
Uptake of PTEs (root and shoot) in the roots and shoots of the leafy vegetables studied, which were grown on a substrate consisting of PTE-contaminated soil–zeolite mixtures in proportions of 0 (control), 50 g zeolite/kg soil (5%) and 100 g zeolite/kg soil (10%).

The concentration of PTEs varies widely between plant species and between roots and shoots (Figure 3). Generally, the roots contain higher concentrations of PTEs than the shoots, except for spinach, where the concentration of Zn is almost twice as high in shoots compared to roots. The higher concentration of Cd in roots than in leaves was reported for *Athyrium esculentum*, *Chromolaena odorata* and *Lantana camara* grown along the main roadside in the town of Jengka, Malaysia [30]. Lower Pb concentration in shoots than in roots was also reported in *Brassica campetris* and *Brassica oleracea* grown in a Pb-contaminated soil [36]. The decreasing microelement concentration in cabbage grown on zeolite-amended sandy soils was reported by Sindesi et al. [37].

The lettuce roots contain the highest concentration of PTEs, except for Cu which has comparable concentration in the roots of spinach and lettuce and is slightly lower in those of parsley. Similarly, the lettuce shoots contain the highest concentration of PTEs, except for Cu and Mn which are comparable in the studied leafy vegetable species. Interestingly, the lettuce root accumulates higher concentrations of Zn than spinach and parsley, while the lettuce shoots contain lower concentrations of Zn than the spinach shoots and are comparable to those of parsley. Medynska-Juraszek et al. [1] also reported that parsley accumulated the lowest amounts of PTEs.

The obtained results confirmed that the addition of zeolite amendments substantially decrease the uptake of PTEs by all studied leafy plants. Previously, Gül et al. [38] reported the beneficial effect of zeolite amendment on the nutrient status of lettuce (*Lactuca sativa* var. *capitata*), as well as the increase in head mass and marketable head mass with increasing ratios of zeolite amendment. Our results are in accordance with those of Contin et al. [18], who reported a significant decrease in PTE content in both roots and shoots of ryegrass (*Lolium multiflorum* L.) cultivated in soil from a contaminated area near a former copper smelter that was amended with 10% (*w*/*w*) natural zeolite (the decrease from 5% addition rate was not statistically significant).

According to EU legislation, for spinaches and similar leaves, as well as fresh herbs, the maximum level for Cd is 0.20 mg/kg *ww*, while for Pb in brassica and leaf vegetables it is 0.30 mg/kg *ww* [26,27,28]. The Cd content in the shoots of spinach and lettuce grown on control, 5% and 10% amended substrate exceeds the maximum level. The maximum level is exceeded in the shoots of parsley grown in the control and 5% amended substrate, but drops below the maximum level in the plants grown on 10% amended substrate (0.19 mg/kg *dw*). The maximum level for Pb is exceeded in all plant species in all experiments.

### 3.4. Soil to Plant Transfer

**Figure 4:**
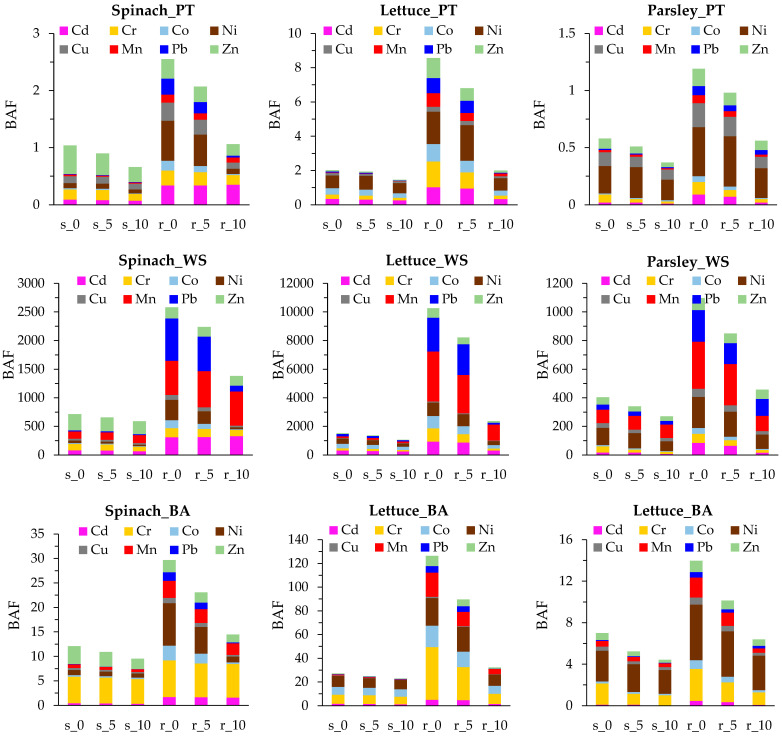
Bioaccumulation factor (BAF) for shoots (s) and for roots (r) calculated for spinach, lettuce and parsley grown in soil–zeolite mixtures in proportions of 0 (control), 50 g zeolite/kg soil (5%) and 100 g zeolite/kg soil (10%) and computed using pseudo total (PT), bioavailable (BA) or water soluble (WS) PTE fractions in soil.

Generally, the BAFr is higher than the BAFs, most probably due to the barrier effect of roots that limits metal uptake as a protective mechanism (Table S1, Figure 4) [31]. However, the BAFs is higher than BAFr for Zn in spinach, probably due to the presence of other protective mechanisms that favor Zn translocation from roots to shoots in order to limit its toxicity.

A slight decrease in both the BAFr and BAFs is observed for the 5% amendment in all species, followed by a sharp decrease for the 10% amendment, indicating that the metal uptake reduction is most efficient for the 10% amendment.

Based on the BAF factors calculated both for accumulation in shoots and roots using the PT PTE concentrations in soils, the studied plant species are neither accumulators (BAF > 1) nor hyperaccumulators (BAF > 10). Similar findings were observed for leafy plants grown in soils containing organic amendment [1]. Root behavior as a barrier against PTEs is well known and may explain its low accumulation. However, by using the WS PTE fraction in soil for calculation of BAF, all three leafy vegetables (spinach, parsley and lettuce) are hyperaccumulators. When considering the BAF of PTEs from soils, parsley may be defined as a Cr and Ni accumulator, spinach as a Cr, Zn and Cd accumulator and lettuce as a Cd, Cr, Cu and Ni accumulator. This fact indicates that, besides the physiological mechanisms of the plant species, varieties and cultivars as well as PTE mobility has an important role in metal uptake and accumulation [1,31,39].

Grown in contaminated soils, lettuce has the highest PTE bioaccumulation capacity, with few exceptions (Zn, Ni), followed by spinach and parsley. This trend is similar for the plants grown in amended soils. Zn is more bioaccumulated in spinach than in parsley and lettuce, while Ni is more bioaccumulated in parsley than in spinach. Therefore, the consumption of leafy vegetables grown on contaminated substrates must be avoided, as it can pose important health risks to humans [7]. Similarly, the reduction in Cd uptake by spinach (*Spinacia oleracea* L.) and lettuce (*Lactuca sativa* L.) after amendment with municipal compost was reported by Krippner et al. [39]. Organic amendment using biochar and a combination of biochar and compost, especially when applied in high doses (10%), also noticeably reduced the uptake of Cu, Zn, Pb, Cd, Cr and Ni by vegetables commonly grown by gardeners, including spinach, lettuce and parsley [1].

**Figure 5:**
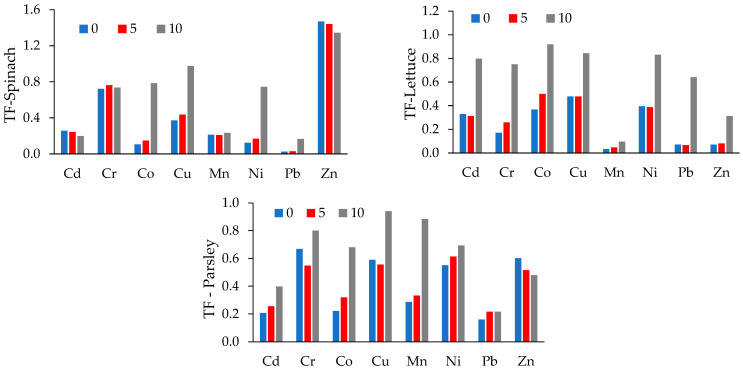
Transfer factors (TF) of PTEs for spinach, lettuce and parsley grown in soil–zeolite mixtures in control (0), 50 g zeolite/kg soil amendment (5%) and 100 g zeolite/kg soil amendment (10%).

Roots to shoots translocation factors (Figure 5, Table S2) in unamended and amended soils are lower than 1, with the exception of Zn in spinach. The TF values for spinach grown on unamended and amended substrates is similar.

Important portions of Zn and Cr are translocated in spinach, Zn, Ni, Cu and Cr in parsley and Cu, Co and Ni in lettuce. These differences may be explained by the different metal sequestration mechanisms of the different species, as well as by the different mobility of the PTEs [30,35]. Generally, the translocation of Co, Cu and Ni increases in spinach with the increase of soil amendment dose. A similar increasing trend is observed for all PTEs in lettuce and for most of the PTEs in parsley. Our findings are similar to those of Krippner and Schubert [39], who reported high shoot to root translocation of Zn in spinach (TF > 4) grown in control soil and reduced translocation (TF < 4) for spinach grown in Zn-contaminated soils. The same study reported that Zn translocation is low, while Cd is retained in the roots of parsley. Oppositely, Cd was found to be retained in the roots of spinach [40].

## 4. Conclusions

The results of pot experiments revealed the capacity of natural zeolite amendment to reduce the accumulation of PTEs in leafy vegetables. The uptake of PTEs from the substrate to plants decreased with the increase of amendment dose, the highest efficiency in metal uptake reduction being observed for the 10% amendment. However, the accumulation in plants depended not only on the amendment dose, but also on the plant species and type, amount, solubility and bioavailability of the PTEs. The accumulation of PTEs was the highest in spinach and the lowest in parsley, but their cultivation in contaminated soils may lead to high content of PTEs, especially in roots and to a lesser extent in the shoots. A notable exception was observed in the case of Zn in spinach, where the concentration in shoots was higher than in roots. By referring to the pseudo total PTE content, lettuce, spinach and parsley did not accumulate nor hyperaccumulate PTEs. However, considering only the water soluble PTE fraction, all species were hyperaccumulators while, considering the bioavailable fraction of the PTEs, parsley accumulated Cr and Ni, spinach Cr, Zn and Cd, and lettuce Cd, Cr, Cu and Ni, respectively. None of the plants displayed root to shoot transfer higher than 1. Further experiments on natural zeolites should include long-term field application on different kinds of soil, different levels of contamination and different types of PTE contamination.

## Figures and Tables

**Table 1:** PTE concentrations expressed as average ± standard deviation (*n* = 3) in the studied leafy vegetable roots and shoots grown in a substrate consisting of PTE-contaminated soil–zeolite mixtures in proportions of 0 (control), 50 g zeolite/kg soil (5%) and 100 g zeolite/kg soil (10%). Different superscript letters represent significant differences (*p* < 0.05) of each PTE and plant species grown in the control, 5% and 10% amended soils.

		Dose (%)	Cd	Cr	Co	Cu	Mn	Ni	Pb	Zn
			mg/kg *dw*							
Spinach	root	0	6.85 ± 0.70 ^a^	3.45 ± 0.41 ^a^	2.84 ± 0.29 ^a^	68.6 ± 4.77 ^a^	305 ± 28 ^a^	10.6 ± 1.6 ^a^	133 ± 13 ^a^	375 ± 28 ^a^
		5	5.94 ± 0.69 ^a^	2.83 ± 0.30 ^a^	1.61 ± 0.21 ^b^	50.6 ± 4.61 ^b^	241 ± 19 ^b^	5.91 ± 0.71 ^b^	96.0 ± 12.6 ^b^	292 ± 26 ^b^
		10	5.60 ± 0.63 ^a^	1.92 ± 0.17 ^b^	0.21 ± 0.02 ^c^	18.8 ± 1.31 ^c^	180 ± 15 ^c^	0.91 ± 0.12 ^c^	14.0 ± 1.6 ^c^	208 ± 18 ^c^
	shoot	0	1.75 ± 0.22 ^a^	2.49 ± 0.16 ^a^	0.30 ± 0.04 ^a^	25.5 ± 1.60 ^a^	64.6 ± 6.6 ^a^	1.31 ± 0.16 ^a^	3.46 ± 0.44 ^a^	550 ± 37 ^a^
		5	1.44 ± 0.14 ^a,b^	2.16 ± 0.19 ^a^	0.24 ± 0.03 ^a^	22.1 ± 1.71 ^a,b^	50.2 ± 4.8 ^b^	1.00 ± 0.12 ^a^	2.91 ± 0.30 ^a,b^	421 ± 30 ^b^
		10	1.11 ± 0.08 ^b^	1.42 ± 0.15 ^b^	0.16 ± 0.02 ^b^	18.3 ± 1.73 ^b^	42.1 ± 3.9 ^b^	0.68 ± 0.1 ^b^	2.32 ± 0.29 ^b^	280 ± 23 ^c^
Lettuce	root	0	20.5 ± 2.05 ^a^	20.4 ± 1.8 ^a^	17.2 ± 1.1 ^a^	63.3 ± 4.71 ^a^	1780 ± 115 ^a^	28.4 ± 3.17 ^a^	425 ± 33 ^a^	1290 ± 76 ^a^
		5	16.5 ± 1.95 ^a^	11.6 ± 0.9 ^b^	10.3 ± 0.7 ^b^	46.0 ± 3.0 ^b^	1020 ± 74 ^b^	22.5 ± 2.8 ^a^	343 ± 26 ^b^	800 ± 50 ^b^
		10	5.30 ± 0.44 ^b^	2.40 ± 0.26 ^c^	4.00 ± 0.36 ^c^	20.4 ± 1.64 ^c^	336 ± 29 ^c^	7.31 ± 0.86 ^b^	18.9 ± 2.5 ^c^	138 ± 13 ^c^
	shoot	0	6.74 ± 0.63 ^a^	3.48 ± 0.36 ^a^	6.32 ± 0.47 ^a^	30.2 ± 1.91 ^a^	57.2 ± 6.7 ^a^	11.2 ± 1.2 ^a^	30.2 ± 4.2 ^a^	92.6 ± 11.4 ^a^
		5	5.14 ± 0.36 ^b^	3.00 ± 0.27 ^a^	5.14 ± 0.47 ^b^	22.0 ± 1.57 ^b^	48.0 ± 4.1 ^a^	8.71 ± 0.87 ^b^	22.6 ± 2.8 ^a^	64.1 ± 6.4 ^b^
		10	4.21 ± 0.36 ^b^	1.80 ± 0.20 ^b^	3.67 ± 0.32 ^c^	17.2 ± 1.25 ^c^	32.4 ± 3.2 ^b^	6.08 ± 0.60 ^c^	12.1 ± 1.8 ^b^	43.1 ± 4.3 ^c^
Parsley	root	0	1.85 ± 0.25 ^a^	1.42 ± 0.14 ^a^	0.80 ± 0.10 ^a^	45.0 ± 3.46 ^a^	168 ± 14 ^a^	6.53 ± 0.68 ^a^	39.8 ± 4.0 ^a^	164 ± 15 ^a^
		5	1.22 ± 0.11 ^b^	0.78 ± 0.08 ^b^	0.45 ± 0.07 ^b^	32.4 ± 2.12 ^b^	109 ± 10 ^b^	4.72 ± 0.65 ^b^	23.2 ± 2.9 ^b^	118 ± 11 ^b^
		10	0.27 ± 0.02 ^c^	0.34 ± 0.04 ^c^	0.12 ± 0.01 ^c^	16.7 ± 1.21 ^c^	32.0 ± 3.4 ^c^	2.60 ± 0.41 ^c^	16.6 ± 1.9 ^b^	80.3 ± 7.0 ^c^
	shoot	0	0.38 ± 0.04 ^a^	0.95 ± 0.11 ^a^	0.18 ± 0.03 ^a^	26.6 ± 1.80 ^a^	48.2 ± 4.6 ^a^	3.60 ± 0.49 ^a^	6.36 ± 0.69 ^a^	98.7 ± 9.2 ^a^
		5	0.31 ± 0.03 ^a^	0.43 ± 0.05 ^b^	0.14 ± 0.02 ^a,b^	18.0 ± 1.25 ^b^	36.4 ± 3.8 ^b^	2.90 ± 0.34 ^a^	5.00 ± 0.50 ^b^	61.0 ± 6.1 ^b^
		10	0.11 ± 0.02 ^b^	0.27 ± 0.03 ^b^	0.08 ± 0.02 ^b^	15.7 ± 1.06 ^b^	28.3 ± 3.8 ^b^	1.80 ± 0.24 ^b^	3.59 ± 0.39 ^c^	38.5 ± 3.8 ^c^

## Data Availability

The data that support the findings of this study are available on request from the corresponding author.

## Supplementary Materials

The following supporting information can be downloaded at: https://www.mdpi.com/article/10.3390/ma15165657/s1. Table S1. Bioaccumulation factor for shoots (BAFs) and for roots (BAFr) calculated for spinach, lettuce and parsley grown on soil-zeolite mixtures in proportion of 0 (control), 50 zeolite/kg soil (5%) and 100 g zeolite/kg soil (10%) using the pseudo total (PT), bioavailable (BA) or water soluble (WS) PTE fractions from soil. Table S2. Transfer factors of PTEs for spinach, lettuce and parsley grown in soil-zeolite mixtures in control (0), 50 g zeolite/kg soil amendment (5%) and 100 g zeolite/kg soil amendment (10%).
