# Supplementary material for: Zeolites Reduce the Transfer of Potentially Toxic Elements from Soil to Leafy Vegetables
Source: Materials (Basel). 2022 Aug 17;15(16):5657. doi: 10.3390/ma15165657 (PMC9416071; doi:10.3390/ma15165657)
Supplement: Supplementary file 1 [file materials-15-05657-s001.zip › materials-1826944-supplementary.pdf]

Zeolites Reduce the Transfer of Potentially Toxic Elements from Soil to Leafy Vegetables

Oana Cadar [1], Zamfira Stupar [1], Marin Senila [1], Levente Levei [1,2], Ana Moldovan [1], Anca Becze [1], Alexandru Ozunu[1,2] and Erika Andrea Levei [1,*]

1 INCDO-INOE 2000, Research Institute for Analytical Instrumentation, 67 Donath Street, 400293 Cluj-Napoca, Romania
2 Faculty of Environmental Sciences and Engineering, Babes-Bolyai University, 30 Fantanele Street, 400294 Cluj-Napoca, Romania
* Correspondence: erika.levei@icia.ro

Table S1 Bioacumulation factor for shoots (BAFs) and for roots (BAFr) calculated for spinach, lettuce and parsley grown on soil-zeolite mixtures in proportion of 0 (control), 50 zeolite/kg soil (5%) and 100 g zeolite/kg soil (10%) using the pseudo total (PT), bioavailable (BA) or water soluble (WS) PTE fractions from soil.

	PT						WS						BA					
	BAFs			BAFr			BAFs			BAFr			BAFs			BAFr		
**Dose**	**0**	**5**	**10**	**0**	**5**	**10**	**0**	**5**	**10**	**0**	**5**	**10**	**0**	**5**	**10**	**0**	**5**	**10**
									**Spinach**									
**Cd**	0.09	0.08	0.07	0.34	0.34	0.35	79.8	75.8	65.2	311	313	329	0.43	0.40	0.31	1.69	1.66	1.57
**Cr**	0.18	0.18	0.12	0.26	0.23	0.17	113	108	78.7	157	142	107	5.42	5.27	5.06	7.50	6.90	6.87
**Co**	0.02	0.02	0.01	0.17	0.11	0.02	15.0	13.3	10.3	142	89.6	13.2	0.32	0.30	0.28	2.99	1.99	0.36
**Cu**	0.12	0.12	0.10	0.32	0.26	0.11	31.8	30.7	26.5	86.0	70.3	27.2	0.40	0.35	0.31	1.07	0.81	0.32
**Mn**	0.03	0.02	0.02	0.14	0.11	0.09	127	132	140	598	634	601	0.74	0.59	0.55	3.49	2.81	2.37
**Ni**	0.09	0.09	0.07	0.70	0.55	0.09	43.7	37.0	26.1	354	219	35.1	1.07	0.93	0.87	8.70	5.47	1.17
**Pb**	0.01	0.01	0.01	0.28	0.20	0.03	19.2	18.2	16.6	739	600	100	0.05	0.04	0.03	1.75	1.34	0.21
**Zn**	0.50	0.38	0.26	0.34	0.27	0.20	284	242	226	193	168	168	3.67	3.01	2.12	2.5	2.09	1.58
									**Lettuce**									
**Cd**	0.34	0.30	0.26	1.02	0.95	0.33	306	271	247	932	868	311	1.66	1.43	1.18	5.05	4.6	1.48
**Cr**	0.26	0.24	0.15	1.51	0.94	0.21	158	150	100	928	580	133	7.57	7.32	6.43	44.4	28.0	8.57
**Co**	0.37	0.34	0.26	1.02	0.68	0.29	316	286	230	861	572	250	6.65	6.35	6.23	18.1	13.0	6.78
**Cu**	0.14	0.11	0.10	0.29	0.24	0.12	37.8	30.6	24.9	79	63.9	29.6	0.47	0.35	0.29	0.98	0.70	0.35
**Mn**	0.03	0.02	0.02	0.79	0.47	0.16	112	126	108	3490	2684	1120	0.65	0.56	0.43	20.4	12.0	4.42
**Ni**	0.74	0.81	0.60	1.88	2.08	0.72	375	323	234	947	833	281	9.21	8.07	7.79	23.3	20.8	9.38
**Pb**	0.06	0.05	0.03	0.89	0.71	0.04	168	141	86.4	2359	2144	135	0.40	0.31	0.18	5.58	4.77	0.28
**Zn**	0.08	0.06	0.04	1.16	0.73	0.13	47.7	36.9	34.8	665	460	111	0.62	0.46	0.33	8.60	5.71	1.05
									**Parsley**									
**Cd**	0.02	0.02	0.01	0.09	0.07	0.02	17.4	16.4	6.30	84.1	64.1	15.9	0.09	0.09	0.03	0.46	0.34	0.08
**Cr**	0.07	0.03	0.02	0.11	0.06	0.03	43.1	21.5	15.1	64.5	39.2	18.9	2.06	1.05	0.97	3.09	1.91	1.21
**Co**	0.01	0.01	0.01	0.05	0.03	0.01	8.90	7.90	5.10	40.0	24.9	7.5	0.19	0.18	0.14	0.84	0.55	0.20
**Cu**	0.12	0.09	0.09	0.21	0.17	0.10	33.2	25.0	22.8	56.3	45.0	24.2	0.41	0.29	0.27	0.70	0.52	0.28
**Mn**	0.02	0.02	0.01	0.07	0.05	0.02	94.4	95.8	94.3	329	288	107	0.55	0.42	0.37	1.92	1.28	0.42
**Ni**	0.24	0.27	0.18	0.43	0.44	0.26	120	107	69.3	218	175	100	2.95	2.68	2.31	5.35	4.37	3.33
**Pb**	0.01	0.01	0.01	0.08	0.05	0.04	35.4	31.2	25.7	221	145	119	0.08	0.07	0.05	0.52	0.32	0.25
**Zn**	0.09	0.06	0.04	0.15	0.11	0.08	50.9	35.1	31.0	84.5	67.9	64.8	0.66	0.44	0.29	1.09	0.84	0.61

Table S2. Transfer factors of PTEs for spinach, lettuce and parsley grown in soil-zeolite mixtures in control (0), 50 g zeolite/kg soil amendment (5%) and 100 g zeolite/kg soil amendment (10%).

TF	Spinach			Lettuce			Parsley		
	**0**	**5**	**10**	**0**	**5**	**10**	**0**	**5**	**10**
**Cd**	0.26	0.24	0.20	0.33	0.31	0.80	0.21	0.26	0.40
**Cr**	0.72	0.76	0.74	0.17	0.26	0.75	0.67	0.55	0.80
**Co**	0.11	0.15	0.78	0.37	0.50	0.92	0.22	0.32	0.68
**Cu**	0.37	0.44	0.97	0.48	0.48	0.84	0.59	0.56	0.94
**Mn**	0.21	0.21	0.23	0.03	0.05	0.10	0.29	0.33	0.88
**Ni**	0.12	0.17	0.74	0.40	0.39	0.83	0.55	0.61	0.69
**Pb**	0.03	0.03	0.17	0.07	0.07	0.64	0.16	0.22	0.22
**Zn**	1.47	1.44	1.34	0.07	0.08	0.31	0.60	0.52	0.48
